# Supplementary figures and images for: The Use of Anaplastic Lymphoma Kinase Inhibitors in Non-Small-Cell Lung Cancer Treatment—Literature Review
Source: Biomedicines. 2024 Oct 11;12(10):2308. doi: 10.3390/biomedicines12102308 (PMC11504905; doi:10.3390/biomedicines12102308)

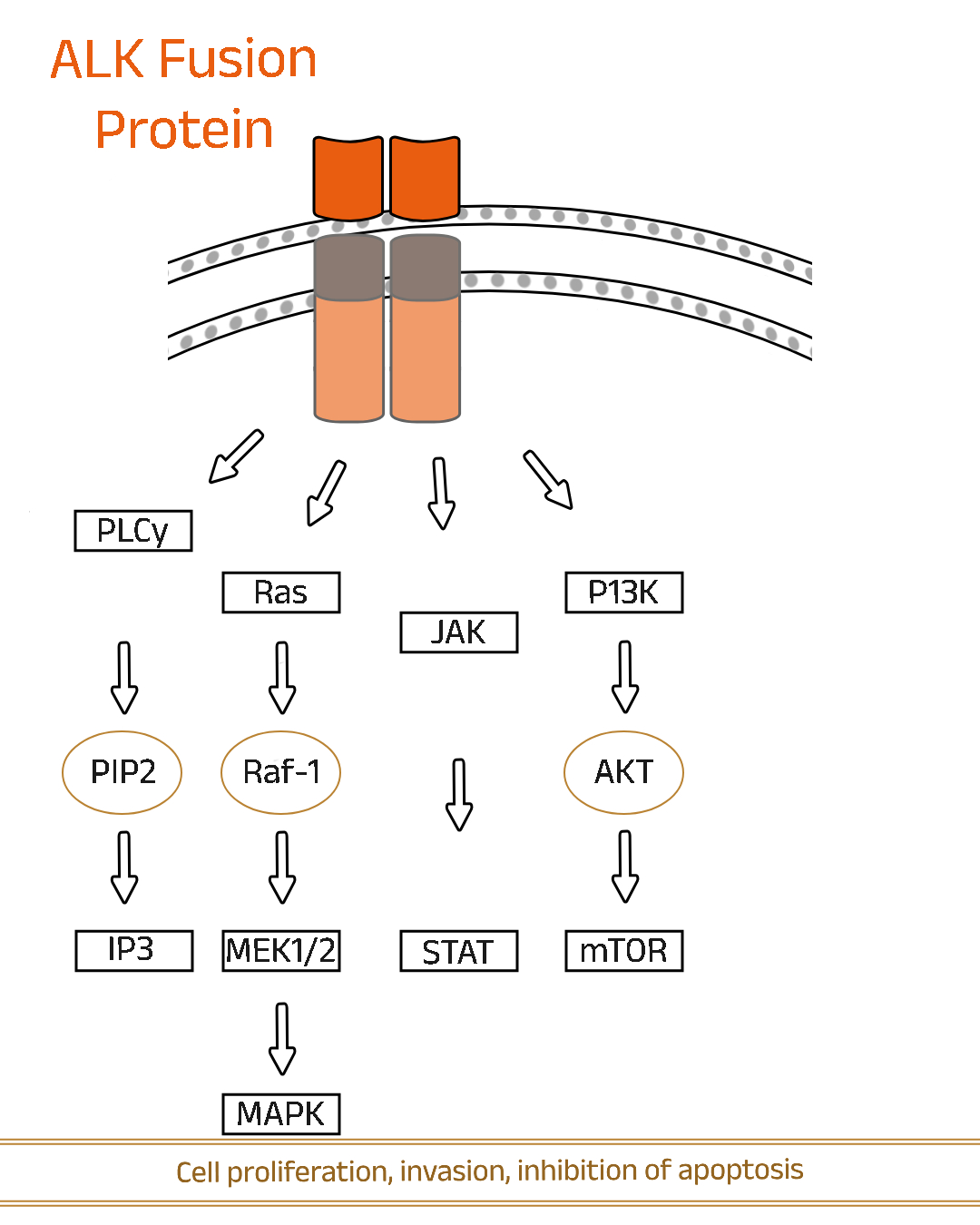

Supplement: Supplementary file 1 [file biomedicines-12-02308-s001.zip › Figure S1.jpg]

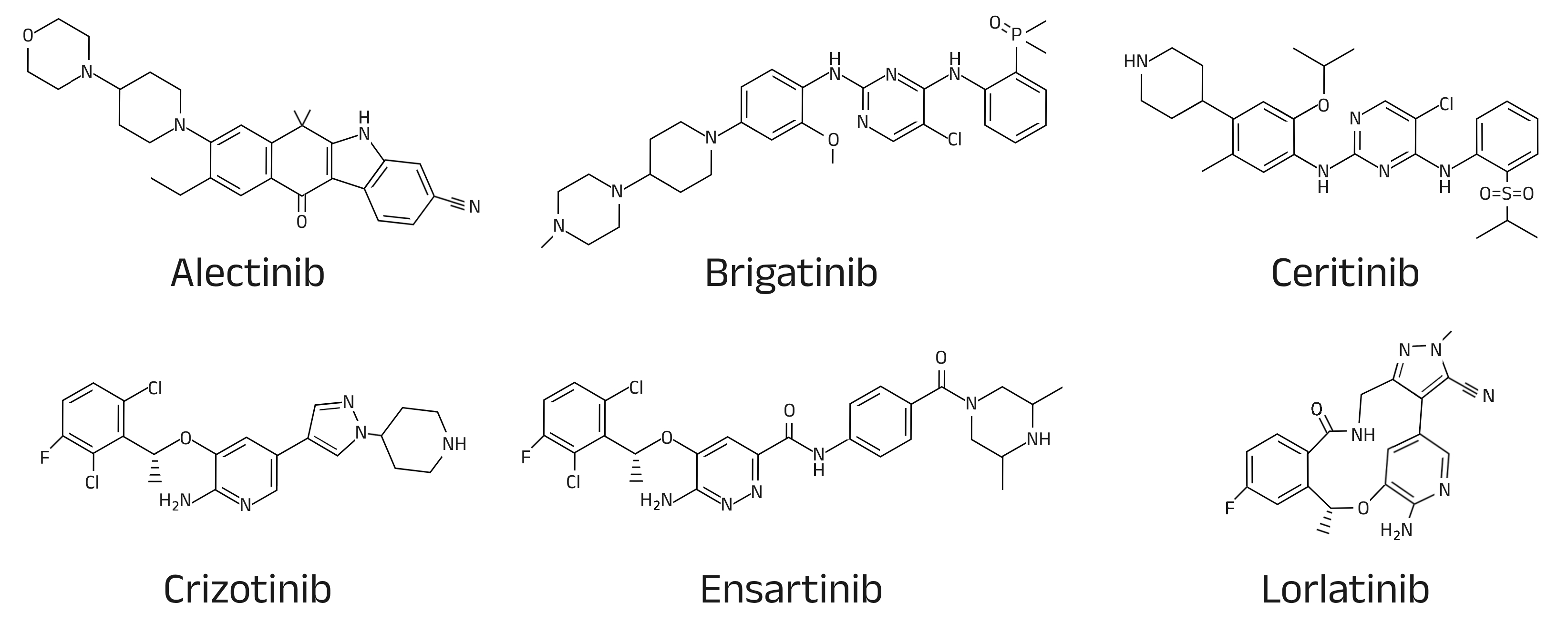

Supplement: Supplementary file 1 [file biomedicines-12-02308-s001.zip › Figure S2.jpg]
